# Risk of SARS-CoV-2 infection and COVID-19 prognosis with the use of renin–angiotensin–aldosterone system (RAAS) inhibitors: a systematic review

**DOI:** 10.1186/s43094-021-00224-4

**Published:** 2021-03-24

**Authors:** Chinonyerem O. Iheanacho, Valentine U. Odili, Uchenna I. H. Eze

**Affiliations:** 1 Department of Clinical Pharmacy and Public Health, Faculty of Pharmacy, University of Calabar, Calabar, Nigeria; 2 Department of Clinical Pharmacy and Pharmacy Practice, Faculty of Pharmacy, niversity of Benin, Benin City, Nigeria; 3 Department of Clinical Pharmacy and Biopharmacy, Faculty of Pharmacy, Olabisi Onabanjo University, Sagamu, Nigeria

**Keywords:** SARS-CoV-2, COVID-19, Prognosis, Hypertension, Cardiovascular diseases, Angiotensin-converting enzyme inhibitors (ACEI), Renin–angiotensin–aldosterone system (RAAS), Angiotensin-2 receptor blockers (ARB)

## Abstract

**Background:**

Angiotensin-converting-enzyme-2, being the receptor for SARS-CoV-2, is increased in the use of RAAS inhibitors. Therefore, concerns have been raised over risks of SARS-CoV-2 infection and poor prognosis of COVID-19 in persons with prior exposure to these drugs. This study aimed to systematically review available evidence for associations between exposure to RAAS inhibitors with susceptibility to SARS-CoV-2 infection and clinical outcomes in infected persons. It hopes to address the question on the effects of RAAS inhibitors on the risk of COVID-19 and its prognosis.

**Main body:**

Search was conducted in the databases of PubMed, Scopus, Cochrane, Embase and MedRxiv.org from December 2019 to May 31, 2020, using relevant keywords. Additional articles were identified through hand-searching of reference lists. Studies that reported associations between positive tests to COVID-19 and use of RAAS inhibitors, and treatment outcomes of COVID-19 patients who had exposure to RAAS inhibitors were considered eligible. The Newcastle–Ottawa scale was used to assess risk of bias in individual studies. The review was conducted in line with Preferred Regulatory Items for Systematic Reviews and Meta-Analysis (PRISMA) guidelines 2009. From the 952 studies screened and 2 studies from reference hand-searching, 18 were reviewed. Four studies evaluated the risks for SARS-CoV-2 infection among RAAS inhibitors users, and 16 (including 2 of the 4 studies) evaluated the clinical outcomes associated with previous exposure to RAAS inhibitors.

**Conclusion:**

Evidence does not suggest higher risks for SARS-CoV-2 infection or poor disease prognosis in the use of RAAS inhibitors. This suggests the continued use of RAAS inhibitors by patients with existing needs, which supports the position statements of American Heart Association and European societies for Cardiology.

**Supplementary Information:**

The online version contains supplementary material available at 10.1186/s43094-021-00224-4.

## Background

Coronavirus disease 2019 (COVID-19) is a disease resulting from the infection of severe acute respiratory syndrome coronavirus-2 (SARS-CoV-2) which has resulted in a global pandemic, after its origination from Wuhan, China, in December, 2019 [1]. Fever, cough, fatigue and gastrointestinal symptoms appear to be the most common clinical features of COVID-19 [2]. Although fatality appears to be predominantly associated with older age, case distribution and severity is influenced by demographic makeup of a population [2].

Persons with comorbidities, particularly cardiovascular diseases appear to be at higher risks of morbidity and mortality from COVID-19 [3, 4]. Studies suggest hypertension and diabetes to be the most common comorbidities in COVID-19, with higher risk associated with older age [4–6]. Persons with hypertension account for majority of the cases and appear to have severe illness from the disease [4]. Diabetes has also been shown to be a predisposing factor for severe COVID-19 illness, with high mortality [4].

Electrolyte balance and blood pressure are regulated by the renin–angiotensin–aldosterone system (RAAS), which has the ACE/Ang11/ATIR and the ACE2/Ang(1-7)Mas receptor, as its pathways [7]. It is an essential vasoactive system involved in the haemostasis of cardiovascular and kidney functions. During the management of cardiovascular diseases (CVD) and diabetes, RAAS effects are frequently altered by its inhibition by several pharmacological agents such as angiotensin-converting enzyme inhibitors (ACEI), angiotensin 11 receptor blockers (ARB) and aldosterone antagonists [8].

**Fig. 1:**
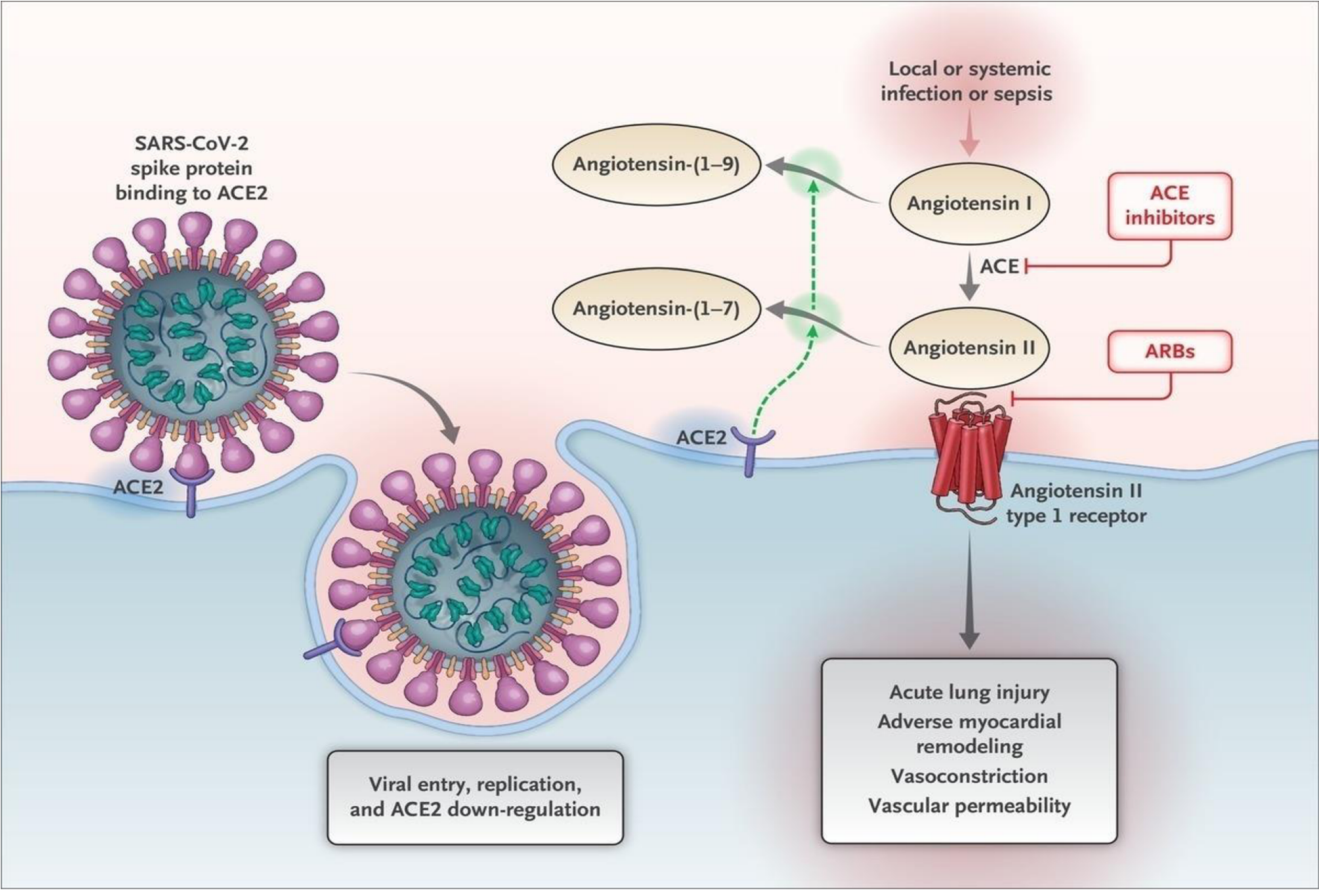
Cell infection of SARS-CoV-2 and the role of renin-angiotensin-aldosterone system [17]

The continued use of renin–angiotensin–aldosterone system inhibitors in persons with cardiovascular diseases and other diseases requiring renal protection has raised safety concerns among several researchers amid the COVID-19 pandemic. This class of drugs has been suggested to upregulate ACE2 [9, 10], which is the receptor for SARS-CoV-2 binding in humans [11], suggesting higher risks of infection and poor prognosis in users [12]. Coronaviruses which are enveloped and single-stranded RNA viruses possess binding affinity for ACE2 human receptors and also possess mechanisms for host cell adaptation [13]. The infection of the ACE2-bearing cells is mediated by the spike (S) protein of the coronaviruses [12, 14]. The spike protein which occurs in two structurally distinct forms as the pre-fusion and post-fusion forms, first binds to the cell surface through its S1 subunit [12]. The S1 domain of the spike protein of SARS-CoV efficiently binds to ACE2 and allows replication of the virus, showing ACE2 to be a functional receptor for SARS-CoV [15]. It has been noted in animal studies that SARS spike protein reduces ACE2 expression, resulting in the promotion of lung injury [11]. This reduction results in an imbalance in the ACE/Ang11/ATIR axis and the ACE2/Ang (1-7)/Mas receptor axis [16] (Fig. 1).

RAAS inhibitors commonly used in the pharmacological management of hypertension may result in increased expression of angiotensin-converting enzyme 2 (ACE2) [9, 10], which is employed by Coronaviruses for cell infection [12], thereby mediating the susceptibility of cells to SARS-CoV and SARS-CoV-2 infections [12, 18]. The observed target cells for SARS-CoV-2 which includes lung alveolar epithelial cells, the kidneys and the heart are made up of broadly expressed angiotensin 2 [19], with higher levels occurring in clinical conditions [20]. They are also largely expressed in the testis and at lower levels in vascular smooth muscle cells [21].

Angiotensin-2 receptor blockers (ARBs) significantly increase cardiac ACE2 levels and circulating angiotensin-2, also ACE inhibitors particularly lisinopril, was observed to increase ACE2 expression in an animal study [10]. Another animal study showed increased expression of ACE2 in the kidneys following the use of an angiotensin 2 receptor blocker [22]. These findings have resulted in concerns that the use of RAAS inhibitors may increase the risk of SARS-CoV-2 infection [23, 24]. Meanwhile, the potential therapeutic effect of Angiotensin-1 receptor (ATIR) blockers in COVID-19 has also been suggested [25], and demographics such as older age and male have been associated with upregulation of ACE2 [26, 27].

RAAS inhibitors are associated with protection of the lungs from severe acute injury in COVID-19, and this is attributed to increased levels of ACE2 [28, 29]. They are also reported to alleviate LPS-induced pneumonia injury [30]. Also, infusion of recombinant ACE2 resulted in a significant prevention of lung injuries in acute respiratory distress syndrome (ARDS) patients [31], which is highly suggestive of the protective role of ACE2 on the lungs in COVID-19. ACE2 is therefore very essential for the protection of the lungs from ARDS, injury following assaults and acute lung failure which is one of the complications associated with COVID-19 [28, 31, 32]. ACE is also involved in immune-modulatory effects as it decreases Th1/Th2 cytokine ratios and inflammatory cytokines in chronic heart failure [33].

This study systematically reviewed the available evidence for increased susceptibility to SARS-CoV-2 infection in persons with previous exposure to RAAS inhibitors,  and the association between RAAS inhibitors and COVID-19 prognosis in infected persons to provide evidence for clinical guidance.

## Main text

**Fig. 2:**
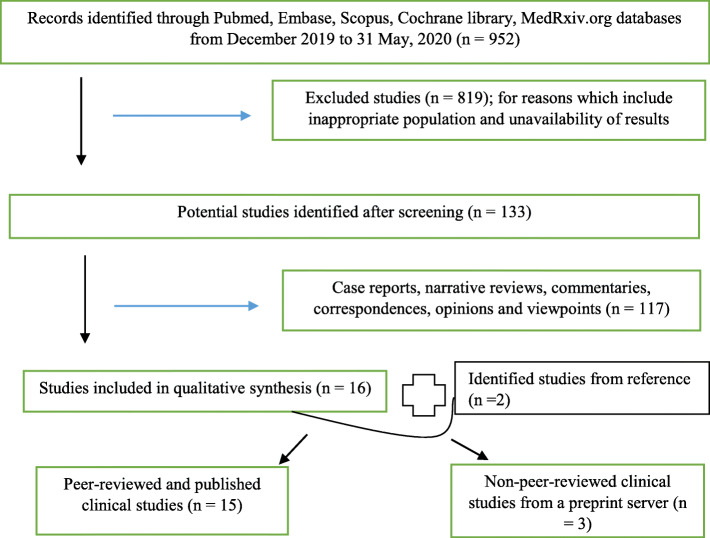
Flowchart showing steps in the qualitative synthesis of evidence from literature

Systematic review of eligible articles, searched from databases of PubMed, Scopus, Cochrane, Embase and MedRxiv.org from December, 2019 up to May 31, 2020, was performed. Keywords used for the search were COVID-19, SARS-CoV-2, Coronavirus diseases 2019, Angiotensin-converting enzyme inhibitors (ACEI), Angiotensin 11 receptor blockers (ARB) and Renin-Angiotensin-Aldosterone System (RAAS). Additional articles were identified through hand-searching of reference lists. Abstracts of the studies were read, and only articles that reported studies on association between COVID-19 and use of RAAS inhibitors were assessed for eligibility. Primary outcomes of interests were increased risk of SARS-CoV-2 infection and worsened COVID prognosis associated with RAAS inhibitors. Original articles and meta-analyses that reported treatment outcomes of adult COVID-19 patients who had exposure to RAAS inhibitors were included in the review, as they provide evidence for the association. Original articles that evaluated the association between exposure to RAAS inhibitors and risk of SARS-CoV-2 infection were also included in the review (Fig. 2). Narrative reviews, commentary, correspondences, and viewpoints were excluded in the study. The studies were in English language and reported the outcomes of interest. The review was conducted in line with Preferred Regulatory Items for Systematic Reviews and Meta-Analysis (PRISMA) guidelines 2009 [34]. Data extraction from reports was performed independently to reduce the risk of bias. Methods employed in the recruitment of study participants, the methods of result analyses and outcome were used to assess risk of bias of individual studies, and this was done using the Newcastle–Ottawa scale for observational studies [35]. See supplementary file for risk of bias assessment tables, s1 and s2.

A total of 952 studies were screened following their relevance to the subject of this review, and 133 were considered eligible. However, 117 articles were letters, narrative reviews, commentaries and case reports, and therefore were not included in the study. A total of 16 studies from the databases and 2 handpicked studies from references were therefore included in the review. Four studies evaluated the risks for testing positive to COVID-19 among RAAS users, and 16 (including 2 of the 4 studies earlier mentioned) evaluated the risks for increased severity and mortality associated with RAAS therapy in COVID-19 patients.

### Relationship between positive tests for SARS-CoV-2 and use of RAAS inhibitors

Findings from a retrospective single-centre study in Florida, USA [36] involved a cohort of 18,472 persons who were tested for COVID-19, among which 12.5% took ACEI/ARB. The retrospective data was collected between March 8 and April 12, 2020. Findings from the study showed 1735 persons tested positive, and no significant relationship was found between use of ACEIs/ARBs and a positive test to SARS-CoV-2 infection, after overlap propensity score weighting. However, the study also found the risk of severe and critical illness to be associated with the use of ACEIs and ARBs, respectively. This however requires further evaluation among a randomized, larger population for a more objective conclusion. Although propensity scores were used to reduce bias, additional cofounders which would be better controlled in a randomized study may have affected the results. Again, potential eligible persons may have been excluded from COVID-19 testing based on the testing criteria of the region; and this will not allow for generalization of the findings as selection was based on tested individuals.

Similarly, another retrospective study by Renyolds et al. observed 5892 COVID-19-positive cases in New York [37] from a study population of 12,594 and did not find any significant association between COVID-19 infection and previous therapy with antihypertensive medications, including ACEIs and ARBs. However, persons on beta blockers were observed to have slightly significantly reduced occurrence of positive tests in the study. Data used for the study were obtained from health records of March 1 to April 15, 2020. The antihypertensive medications also did not appear to be significantly associated with the risk of severe illness among the patients. Several limitations of the study, among which are the study design and actual sensitivity of testing methods employed, will warrant further study for evaluation. Also, data on medication history received directly from users or caregivers of COVID-19 patients may provide more current and appropriate information than electronic health record as used in the study. Although propensity score was used in data analysis, unmeasured cofounding is not unlikely.

Also, Rentsch et al. reported lack of significant association between testing positive to COVID-19 and exposure to ACEI/ARBs among older population of 54–75 years old in the USA [38]. The researchers retrospectively evaluated a healthcare record from February 8 to March 30, 2020 from a population of 2,026,227. The study was not peer-reviewed as at the time of this review, but provides potential evidence to allay fears and confusion on ACE/ARB therapy in the course of COVID-19 pandemic. ARBs/ACEIs were not reported to be associated with COVID-19-positive tests; however, age, race and sex, among others, were associated with risk of positive test.

In a case–population study in Lombardy, Italy, Mancia et al. evaluated the risk of RAAS inhibitors and COVID-19 [39]. In the retrospective study, 6272 cases were matched to 30,759 population from February 21 to March 11, 2020. Although the use of ACEIs and AREBs was more common in the COVID-19 cases, it was observed that the use of ARB and ACEI was not associated with COVID-19 among the patients (95% CI, 0.86 to 1.05) and 0.91 (95% CI, 0.87 to 1.07), respectively. Potential for cofounders is associated with the study.

The above four studies are observational and therefore possess the inherent limitations of observational studies but provide evidence that does not suggest increased risk of contracting COVID-19 in the use of ACEIs/ARBs.

### Prognosis of COVID-19 in patients on therapy with RAAS inhibitors

The use of ARBs/ACEIs may facilitate SARS-COV-2 infection and replication, but the clinical outcomes associated with it are not certain. For adequate and appropriate clinical decisions, several studies are being conducted to assess and determine the effects of RAAS inhibitors on risks for severe COVID-19 illness and prognosis. Several randomized clinical trials have also been proposed to enhance the clinical knowledge in this area of focus. Among these is a randomized clinical trial with NCT04338009 identifier, which hopes to assess the effects of RAAS on COVID-19 severity by assessing the outcomes of COVID-19 associated with continuation and discontinuation of ARBs and ACEIs [40]. With the unavailability of sufficient evidence to consider the risks of RAAS inhibitor therapy in COVID-19; the Council of Hypertension of the European Society of Cardiology has advised the continued use of the drugs [41, 42].

Contrary to the hypothesis of poor clinical outcomes associated with ACEI/ARB use in COVID-19, a study by Abajo et al., conducted in Madrid, Spain [43] involving 1139 cases and 11,390 control COVID-19 patients in multicenter, reported that the use of RAAS was not associated with increased risk of COVID-19 illness and hospital admission (adjusted OR = 0.80, 0.64 to 1.00 and 1.10, 0.88 to 1.37 for ACEI and ARB, respectively). A total of 43.6 and 33.6% used RAAS inhibitors among the case and the control groups, respectively. The duration of study was from March 1 to March 24, 2020, and findings suggest protection from severe COVID-19-associated illness among diabetes patients who used RAAS inhibitors (adjusted OR = 0.53, 95% CI, 0.34 to 0.80). The multicentre design and large sample size add strength to the study, and findings are reassuring but require further studies for evaluation.

Similarly, another study conducted in London, UK [44] found the use of ACE inhibitors to be associated with reduced risk of COVID-19 severity and mortality. The study noted that only 14% of COVID-19 patients with previous exposure to ACE inhibitors either died or had critical illness as compared with 29% of patients without a history of ACEI use. This finding may be explained by the reported protective effects of RAAS inhibitors from severe acute lung injury in COVID-19. Patients who had ACEI/ARBs exposure were significantly older and had more comorbidities than others in the cohort. Data for the study were retrospectively collected from March 1 to March 22, 2020. The study, however, consisted of a small sample size of 205 patients drawn from two centres and, therefore, may not be an accurate representation of the larger population.

De Spiegeleer et al. [45] found no significant relationship between the use of ACEIs/ARBs and absence of symptoms in COVID-19 (OR 0.75; CI 0.25–1.85; *p* = 0.556) (Table 1). The researchers also found that ACEI/ARB use was not significantly associated with serious clinical outcomes of COVID-19 (OR 0.79; CI 0.26–1.95; *p* = 0.629). The researchers however, advised caution in interpreting the lack of statistical significance, sighting the small sample size as a possible factor. The retrospective study was conducted among 154 patients in 2 nursing homes in Belgium, and data were collected from March 1 to April 16, 2020. The analysis included adjustment for covariates which were age, sex, comorbidities and functional status. Although the study comprised a small sample size, it draws attention to the effects of ACEIs/ARBs on the older population with COVID-19.

**Table 1:** Characteristics of the studies

Study	Sample size	Study design	Outcomes	Limitations
Mehta et al. 2020 [36], records from March 8 to April 12, 2020	1735	Retrospective	No association between positive tests to COVID-19 and use of ACEI/ARB	Small sample size, inherent bias from observational studies
Reynolds et al. 2020 [37], data from March 1 to April 15, 2020	5892	Retrospective	No association between risk of positive tests to COVID-19 and risk of severe illness with use of ARB/ACEI	Small sample size, potential confounder bias
Rentsch et al. 2020 [38], data from February 8 to March 30, 2020	585	Retrospective	Encouraged continued use of ACEI/ARBs. Drugs not associated with need for intensive care	Not yet peer-reviewed (as at the time of the study)
Mancia et al. 2020 [39], records from February 21 to March 11, 2020	6272	Retrospective	ARB/ACEI use not associated with increased risk of contracting COVID-19, severity and mortality from the disease	Study design was not randomised
Abajo et al. 2020 [43], health records from March 1 to March 24, 2020	1139	Retrospective case–control	ARBs/ACEIs use did not increase risk for hospitalization in COVID-19 patients	Possible confounders
Bean et al. 2020 [44], data from March 1 to March 22, 2020	205	Observational (prospective)	ARBs/ACEIs appeared to reduce morbidity and mortality in COVID-19	Small sample size, single-centre, short follow-up, not peer-reviewed (as at the time of this study)
De Spiegeleer et al. 2020 [45], health record from March 1 to April 16, 2020	154	Retrospective	ARB/ACEI use was neither associated with absence of COVID-19 symptoms nor serious clinical outcomes	Small sample size
Li et al. 2020 [46], records from January 15 to March 15, 2020	1178	Retrospective	ACEI/ARB use was not significantly associated with severity and mortality from COVID-19	Single-centre study, not randomised
Liu et al. 2020 [47], records from December 29, 2019 to February 29, 2020	511 elderly	Retrospective	Drugs associated with decreased disease severity	Small sample size of understudied groups, not peer-reviewed (as at the time of the study)
Meng et al. 2020 [48], records from January 11 to February 23, 2020	42	Retrospective	Lower levels of IL-6 and reduction in viral load, with the use of ACEIs/ARBs	Very small sample size
Rossi et al. 2020 [49], health records from February 22 to April 2, 2020	2653	Retrospective	Study drugs not associated with risk of mortality	Potential for confounders
Yang et al. 2020 [50], data from January 5 to February 22, 2020	2068	Retrospective	Lower case of critical illness and mortality in ARB/ACEI users	Single-centre, potential confounders
Feng et al. 2020 [51], data from January 1 to February, 15, 2020	476	Retrospective	Use of ACEIs/ARBs appeared to lower risks of severe COVID-19 illness	Potential confounders, small sample size
Zhang et al. 2020 [52], data from December 31 2019 to February 20, 2020	1128	Retrospective	ACEI/ARB use associated with lower mortality	Potential confounders
Peng et al. 2020 [53], health records January 20 to February 15, 2020	112	Retrospective	ACEIs/ARBs not associated with critical illness and mortality in COVID-19	Small sample size
Huang et al. 2020 [54], data from February 7 to March 3, 2020	50	Retrospective	No significant difference in disease course in the use of ACEIs/ARBs and other classes of antihypertensive	Small sample size
Zhang et al. 2020 [55], clinical data up to May 9, 2020	14 studies	Meta-analysis	ARBs/ACEIs not associated with higher risk of COVID-19 infection, severity and mortality	Potential confounders, small number of eligible studies
Guo et al. 2020 [56], clinical data up to May 13, 2020	9 studies	Meta-analysis	ARB/ACEI use not associated with increase severity of COVID-19	Potential confounders

Findings from a retrospective study in Wuhan, China [46] also encourages the continued use of ACEIs and ARBs in COVID-19. The single-centre study which involved 1178 COVID-19 patients found no significant association between patients with current exposure to ACEIs and ARBs with patients without such exposure. Data from January 15 to March 15, 2020 collected for the study showed that out of the 362 (30.7%) patients who had hypertension, 115 (31.8%) were managed with ACEI/ARBs. It also noted that the ACEI/ARBs were not associated with severity and mortality from COVID-19. There was no significant difference between ACEI/ARB users in the groups with severe and non-severe disease (32.9% vs 30.7%; *p* = 0.65), with “survivours and non-survivours” (27.3% vs 33.0%; *p* = 0.34). The duration of retrospective data collection was moderate, but the study was carried out in a single centre. Therefore, replications with a randomized study design in a larger population will provide better evaluation of these associations. It is noteworthy that patients who were exposed to ACEIs/ARBs were not comparably matched with others in this study.

Furthermore, another retrospective study conducted in China [47], among 511 COVID-19 patients with focus on the elderly (> 65 years old), observed ARB use prior to hospitalization to be significantly associated with decrease in severity as compared with patients who had no history of antihypertensive drug therapy. Data from patients who were admitted between December 29, 2019 and February 29, 2020 were analyzed for the study. Other antihypertensive drug groups also studied were persons who took calcium channel blockers, beta blockers, thiazides and ACE inhibitors. The researchers also performed a meta-analysis using 3 previous studies, and findings suggest that ACEIs/ARBs may be associated with decreased pneumonia-related mortality. Although, encouraging findings are seen in this study particularly for the elderly, it should be noted that the potential for bias from cofounders is not unlikely. Also, analysis for significant differences was not done for some groups of antihypertensive drug users due to small sample size.

Another study in the USA [38] involved a much larger, older population and reported the absence of significant relationship with the use of ACEI/ARB and hospitalization/intensive care in COVID-19. Data from a secure health record was collected retrospectively for a cohort of 2,026,227, from which 585 (15.4% of 3789 persons who tested positive) COVID-19 cases of 54–75 years old were studied. ACE/ARB users made up 40.5% of studied cases. These findings are suggestive of the continued use of these classes of drugs for clinical needs, even in high-risk COVID-19 communities. Meanwhile, the design of this study and its findings were yet to be evaluated by peer review as at the time of this review.

Again, an observational study in Shenzhen, China by Meng et al. suggests beneficial effects of ARBs and ACEIs in the clinical outcomes of COVID-19 [48]. The retrospective study noted lower rates of severe COVID-19 illness and lower levels of IL-6 in patents who took ARBs or ACEIs. The findings were from clinical data of patients admitted between January 11 and February 23, 2020. Findings suggest enhanced immunity in patients who were on ACEI or ARB therapy, as increased CD3 and CD8 T cell counts were noted in this group. Viral load was also reportedly reduced in the ACEI/ARB group, as compared with the control group where other classes of antihypertensive drugs were used. Meanwhile, from a total of 417 COVID-19 patients drawn for the study, only 42 patients represented the ACEI and ARB group, constituting a small number of clinical cases. Therefore, findings cannot be extrapolated to the larger population.

In an Italian population-based study that comprised 2653 COVID-19 patients, it was observed that previous therapy with ACEI did not show association with risk of mortality (HR 0.97, 95% CI 0.69 to 1.34) [49]. The study which was aimed at understanding the factors that influence the natural course of COVID-19, generated its data from archived data and involved only symptomatic patients. Researchers retrospectively observed a cohort from a health record data from February 27 to April 2, 2020. Age and comorbidities were statistically adjusted in the analysis of the results.

Again, a retrospective single-centre study [50] among 126 COVID-19 patients with pre-existing hypertension, of which 43 were treated with ACEI or ARB, evaluated the association between ACEI/ARB use and clinical outcome of COVID-19. A group of 125 non-hypertensive patients were included as control for the study. The patients were observed from January 5 to February 22, 2020. A population of 1942 hypertensive, non-COVID-19 patients were included for external control. In this Chinese study, the ARB/ACEI group were observed to have lower cases of critical illness and lower mortality than the control group, but the difference was not statistically significant. The drugs were suggested to have exerted anti-inflammatory effects and improved clinical outcome of the patients. These findings are suggestive of positive association between RAS inhibitors and COVID-19 natural course, but were yet to be validated by peer review during the review period.

In a study by Feng et al., a significant difference in the use of ACEI/ARBs and disease severity was reported, with a higher percentage of patients who had moderate illness using ACEIs/ARBs (87.5 and 85.2% respectively) [51]. The retrospective study included 476 patients who were drawn from 3 hospitals in China, and clinical data was collected from January 1 to February 15, 2020. Although cofounders were not accounted for in the analysis and it was mostly descriptive, it suggests safety of RAAS therapy in COVID-19.

A retrospective study with data drawn from 9 hospitals in Hubei, China [52] included 1128 adult COVID-19 patients with history of hypertension, of which 188 persons where on ACEI/ARB therapy. The clinical data were drawn from December 31, 2019 to February 20, 2020. The median age of the group was 64, and interquartile range was 55–68, while the non-ACEI/ARB group had a median age of 64 and interquartile range of 57–69. The ACEI/ARB group was made up of 188 patients, while the control group was composed of 940 COVID-19 patients who did not take ACEI/ARB. Findings from the study showed significantly lower mortality rate in the ACEI/ARB group, in unadjusted mortality rate (*p = 0.01*), which was consistent after adjusting for cofounders for all-cause mortality (*p = 0.03*), and in propensity score analysis (*p = 0.03*). Potential residual cofounders may not be completely ruled out, hence the need for a case–control validation of findings.

Peng et al. [53] observed no significant difference in the association between the use of ACEI/ARB and critical disease in COVID-19, as well as its use and COVID-19-related mortality, in patients with co-existing cardiovascular diseases. The retrospective study involved 112 persons who were diagnosed of COVID-19 in a hospital in Wuhan, China between January 20 and February 15, 2020. The study population was divided into critical group (ICU = 16) and general group (*n* = 96), with the critical group having a significantly higher BMI (*p = 0.003*). The study however, utilised a small sample size.

Another study in Wuhan, China [54] observed that there was no significant difference in the clinical outcomes (severity, clinical course and mortality) of ACEI/ARB therapy in COVID-19 and therapy with other classes of antihypertensive, among COVID-19 hypertensive patients. Using data from a health record, the researchers retrospectively studied 50 COVID-19 patients with hypertension from February 7 to March 3, 2020. The study group was made up of 20 patients who were treated with ACEI/ARB, while the control group was made up of 30 patients who were treated with other classes of antihypertensive drugs. The antihypertensive drugs were not withdrawn during therapy for COVID-19. The researchers noted that serum cardiac troponin I (cTnI) and N-terminal pro hormone (NT-proBNP) were significantly lower in the ACEI/ARB group (*p = 0.03* and *p = 0.04* respectively), but this was age dependent. The participants in the ACEI/ARB group were younger than the group whose hypertension was managed by other classes of antihypertensive drugs (mean age = 52.65 ± 13.12 and mean age = 67.77± 12.84 respectively; *p = 0.000*), and this may have influenced the findings. The small sample population also poses several limitations to the study and its findings. The study only focused on inpatients; hence, the outcomes in the milder cases were not included.

The previously reported population–case–control study conducted in the Lombardy region of Italy in 6272 confirmed COVID-19 patients also evaluated the effects of ARB/ACEI on disease severity and mortality [39]. After matching the patients to 30,759 health service users in the region, ACEIs/ARBs was also reported not to be associated with severity and mortality from COVID-19, adjusted odds ratio for ARB 0.83 (95% CI, 0.63 to 1.10) and adjusted ratio for ACEI 0.91 (95% CI, 0.69 to 1.21). More persons with exposure to ACEI and ARB were found among the cases than the control, and the researchers attributed this to higher prevalence of cardiovascular conditions in the cases.

Similarly, a meta-analysis [55] of 14 articles that composed of more than 19,000 cases of COVID-19 showed that exposure to ACEI/ARB was not associated with higher risks for COVID-19 infection (OR = 0.99; 95% CI, 0.95–1.04; *p* = 0.672). ACEI/ARB therapy was also not found to be associated with increased severity and mortality in COVID-19 patients (OR = 0.98; 95% CI 0.87–1.09; *p* = 0.69 and OR 0.73, 95% CI 0.5–1.07; *p* = 0.111, respectively). Meanwhile, significantly lower risk of mortality was associated with the use of ACEI/ARB use as compared with other antihypertensive medications (OR = 0.48, 95% CI 0.29–0.81 *p* = 0.006). Relevant article up to May 9, 2020 were used in the analysis. The potentials for selection bias and effects of cofounding socio-demographics in the individual studies, which may influence the reliability of results, may not be overlooked.

Another meta-analysis included 9 studies and a total of 3936 COVID-19 patients who were previously diagnosed of hypertension [56]. Eligible studies up to May 13, 2020 were included in the study. The researchers noted that exposure to ACEI/ARB was not associated with increased COVID-19 severity (OR = 0.71; 95% CI 0.46–1.08 *p* = 0.11) but was significantly associated with lower mortality rate (OR = 0.57; 95% CI 0.38–0.84; *p* = 0.004). It is imperative to note that selection bias may also influence the accuracy of these reports.

This study has enhanced evidence showing the insignificant association between SARS-CoV-2 and COVID-19 with RAAS inhibitors. This is relevant in providing basis for relevant counsel by health care providers during this pandemic. Meanwhile, several limitations were associated with the individual studies, one of which was the use of small sample size in a majority of them which may not be reflective of the larger populations. The majority of the studies also had high potentials for cofounder bias, inherent in observational studies, following the predominant retrospective study design. Some of the articles were also pre-prints, which were yet to be validated by peer review and hence are not to be relied for medical decisions. Findings from case–control prospective randomized clinical trials with large sample, will be more representative of the true associations and provide more objective conclusions. Also, incomplete reporting may not be completely ruled out in this review which may influence the accuracy of findings. Only 5 databases were searched for eligible articles, and this may likely pose risk of selection bias.

## Conclusion

Despite potential enhanced expression of ACE2 during therapy with RAAS inhibitors, available evidence shows no significant associations between exposure to RAAS inhibitors and susceptibility to COVID-19, as well as poor disease prognosis. The results show that RAAS therapy may not be associated with higher risks for contracting COVID-19, higher risks of disease severity and mortality from COVID-19. Associations that relate to improved clinical outcomes were rather seen, suggesting beneficial effects of its continued use among patients with clinical needs, regardless of COVID-19.

This finding therefore supports the position statements of the American Heart Association and the European Societies of Cardiology on the continued use of RAAS inhibitors in COVID-19 patients and persons in high-risk communities, except medically recommended otherwise.

## Supplementary Information

**Additional file 1.** Assessment of risks of bias for observational studies according to the Newcastle–Ottawa scale.

## Data Availability

Not applicable.

## Abbreviations

SARS-CoV-2: Severe acute respiratory syndrome coronavirus 2
COVID-19: Coronaviruses disease 2019
RAAS: Renin–aldosterone–angiotensin system
ACEI: Angiotensin-converting enzyme inhibitors
ARBs: Angiotensin receptor blockers
ACE: Angiotensin-converting enzyme
ACE2: Angiotensin-converting enzyme 2

## References

[1] Zhu N, Zhang D, Wang W, Li X, Yang B, Song J, Zhao X, Huang B, Si W, Lu R, Niu P, Zhan F, Ma X, Wang D, Xu W, Wu G, Gao GF, Tan W (2020). A novel coronavirus from patients with pneumonia in China. N Engl J Med.

[2] Onder G, Rezza G, Brusaferro S (2020) Case fatality rate and characteristics of patients dying in relation to COVID-19 in Italy. JAMA. 10.1001/jama.2020.4683 10.1001/jama.2020.4683 32203977

[3] Huang C, Wang Y, Li X, Ren L, Zhoa J, Hu Y, Zhang L, Fan G, Xu J, Gu X, Cheng Z, Yu T, Xia J, Wei Y, Wu W, Xie X, Yin W, Li H, Liu M, Xiao Y, Gao H, Xie J, Wang G, Jiang R, Gao Z, Jin Q, Wang J, Cao B (2020). Clinical features of patients infected with 2019 novel coronavirus in Wuhan, China. Lancet.

[4] Wang D, Hu B, Hu C, Zhu F, Liu X, Zhang J, Wang B, Xiang H, Cheng Z, Xiong Y, Zhao Y, Li Y, Wang X, Peng Z (2020). Clinical characteristics of 138 hospitalized patients with 2019 novel coronavirus infected pneumonia in Wuhan, China. JAMA.

[5] Zhang JJ, Dong X, Cao Y, Yuan Y, Yang Y, Yan Y, Akdis CA, Gao Y (2020). Clinical characteristics of 140 patients infected by SARS-CoV-2 in Wuhan, China. Allergy.

[6] Zhou F, Yu T, Du R, Fan G, Liu Y, Liu Z, Xiang J, Wang Y, Song B, Gu X, Guan L, Wei Y, Li H, Wu X, Xu J, Tu S, Zhang Y, Chen H, Cao B (2020). Clinical course and risk factors for mortality of adult in-patients with COVID-19 in Wuhan, China; a retrospective cohort study. Lancet.

[7] Hampl V, Herget J, Bibova J (2015). Intrapulmonay activation of angiotensin-converting enzyme type 2/angiotensin 1-7/G-protein-coupled Mas receptor axis attenuates pulmonary hypertension in Ren-2 transgenic rats exposed to chronic hypoxia. Physiol Res.

[8] Fountain JH, Lappin SL (2020). Physiology, renin-angiotensin system (updated 2019 May,5). StatPearls (Internet).

[9] Hoffman M, Kleine-Weber H, Schroeder S, Kruger N, Herrler T, Enrishsen S, Schiergens TS, Herrler G, Wu N, Nitsche A, Muller MA, Drosten C, Pohlmann S (2020). SARS-CoV-2 cell entry depends on ACE2 and TMPRSS 2 and is blocked by clinically proven protease inhibitor. Cell..

[10] Ferrario CM, Jessup J, Chappell MC, Adverill DB, Brosnihan KB, Tallant A, Diz DI, Gallagher PE (2005). Effects of angiotensin-converting enzyme inhibition and angiotensin 11 receptor blockers on cardiac angiotensin-converting enzyme 2. Circulation.

[11] Glowacka I, Bertram S, Herzog P, Pfefferle S, Steffen I, Muench MO, Simmons G, Hofmann HKT, Weber F, Eichler J, Drosten C, Pohlmann S (2010). Differential downregulatin of ACE2 by spike proteins of severe acute respiratory syndrome coronavirus and human coronaviruses NL63. J Virol.

[12] Zhou P, Yang XL, Wang X, Hu B, Zhang L, Zhang W, Si HR, Zhu Y, Li B, Huang CL, Chen HD, Chen J, Luo Y, Guo H, Jiang RD, Liu MQ, Chen Y, Shen XR, Wang X, Zheng XS, Zhao K, Chen QJ, Deng F, Liu LL, Yang B, Zhan FX, Wang YY, Xiao GF, Shi ZL (2012). A pneumonia outbreak associated with a new coronavirus of probable bat origin. Nature.

[13] Wu K, Peng GQ, Wiken M, Geraghty RJ, Li F (2012). Mechanism of host receptor adaptations by severe acute respiratory syndrome virus. J Bio Chem.

[14] Moore MJ, Dorfaman T, Li W, Wong SK, Li Y, Kuhn JH, Coderre J, Vasilieva N, Han Z, Greenough TC, Farzan M, Choe H (2004). Retroviruses pseudotyped with the severe acute respiratory syndrome coronavirus spike protein efficiently infects cells expressing angiotensin converting enzyme 2. J Virol. Doi.

[15] Wenhui L, Moore MJ, Vaslieva N, Sui J, Wong SK, Berne MA, Somasundaran M, Sullivan JL, Luzuriaga K, Greenough TC, Choe H, Farzan M (2003). Angiotensin converting enzyme is a functional receptor for the SARS coronavirus. Nature.

[16] Kuba K, Imai Y, Rao S, Gao H, Guo B, Guan B, Huan Y, Yang P, Zhang Y, Deng W, Bao L, Zhang B, Liu G, Wang Z, Chappell M, Liu Y, Zheng D, Leibbrandt A, Wada T, Slutsky AS, Liu D, Qin C, Jiang C, Penninger JM (2005). A crucial role of angiotensin-converting enzyme 2 (ACE2) in SARS coronavirus-induced lung injury. Nat Med.

[17] Vaduganathan M, Vardeny O, Michel T, JJV MM, Pfeffer MA, Solomon SD (2020). Renin-angiotensin-aldosterone-system inhibitors in patients with COVID-19. New Engl J Med.

[18] Wang P, Chen J, Zheng A, Nie Y, Shi X, Wang W, Wang G, Luo M, Liu H, Tan L, Song X, Wang Z, Yin X, Qu X, Wang X, Qing T, Ding M, Deng H (2004). Expression cloning of functional receptor used by SARS coronavirus. Biophys Res Commun.

[19] Hamming I, Timens W, Bulthuis MLC, Lely AT, Navis GT, van Goor H (2004). Tissue distribution of ACE2 protein, the functional receptor for SARS coronavirus: a first step in understanding SARS pathogenesis. J Pthol.

[20] Serfozo P, Wysocki J, Gulua G, Schulze A, Ye M, Liu P, Jin J, Bader M, Myohanen T, Garcia-Hosman JA, Batle D (2020). Ang II (angiotensin II) conversion to angiotensin-(1-7) in the circulation is POP (prolyloligopeptidase)-dependent and ACE2 (angiotensin-converting enzyme 2)-dependent. Hypertension..

[21] Donoghue M, Hsieh F, Baronas E, Godbout K, Gosselin M, Stagliano N, Donovan M, Woolf B, Robinson K, Jeyaseelan R, Breitbart RE, Acton S (2000). A novel angiotensin-converting enzyme-related carboxypeptidase (ACE2) converts angiotensin 1 to angiotensin 1-9. Cir Res.

[22] Solar MJ, Ye M, Wysocki J, William J, Lloveras J, Batlle D (2009). Localization of ACE2 in renal vasculature: amplification by angiotensin II type 1 blockade using telmisartan. Am J Physiol Renal Physiol.

[23] Sommerstein R, Grani C (2020) Rapid response: re: preventing a COVID-19 pandemic: ACE inhibitors as a potential risk factor for fatal Covid-19. BMJ 368:m810. 10.1136/bmj.m810 10.1136/bmj.m810 32111649

[24] Fang L, Karukiulakis G, Roth M (2020) Are patients with hypertension and diabetes at increased risk for COVID-19 infection. Lancet Resp Med 8(4):e21. 10.1016/S2213-2600(20)30116-8 10.1016/S2213-2600(20)30116-8 PMC7118626 32171062

[25] Gurwitz D (2020). Angiotensin receptor blockers as tentative SARS-CoV-2 therapeutics. Drug Dev Res..

[26] Fernandez-Atucha A, Izagirre A, Fraile-Bermudez AB, Kortajarena M, Larrinaga G, Martinez-Lage P, Eche Varria E, Gil J (2017). Sex differences in the ageing pattern of renin-angiotensin system serum peptidases. Biol sex Differ.

[27] Walters TE, Kalman JM, Patel SK, Mearns M, Vel Koska E, Burrel LM (2017). Angiotensin converting enzyme 2 activity and human arterial fibrillation: increase plasma angiotensin converting enzyme 2 activity in association with arterial fibrillation and more advanced left atria 1 structure remodelling. Europace..

[28] Imai Y, Kuba K, Rao S, Huan Y, Guo F, Guan B, Yang P, Sarao R, Wada T, Leong-Poi H, Crackower MA, Fukamizu A, Hui C, Hein L, Uhling S, Slutsky AS, Jiang C, Penninger JM (2005). Angiotensin-converting enzyme 2 protects from severe acute lung failure. Nature.

[29] Yang P, Gu H, Zhoa Z, Wang W, Cao B, Lai C, Yang X, Zhang L, Duan Y, Zhang S, Chen W, Zhen W, Cai M, Penninger JM, Jiang C, Wang X (2015). Angiotensin converting enzyme (ACE2) mediates influenza H7N9 virus-induced acute lung injury. Sci Rep.

[30] Ye R, Liu Z (2020). ACE exhibits protective effects against LPS-induced acute lung injury in mice by inhibiting the LPS-ILR4 pathway. Exp Mol Pathol.

[31] Khan A, Benthin C, Zeno B, Albertson TE, Boyd J, Christie JD, Hall R, Poirier G, Ronco JJ, Tidswell M, Hardes K, Powley WM, Wright TJ, Siederer SK, Fairman DA, Lipson DA, Bayliffe AI, Lazzar AL (2017). A pilot clinical trial of recombinant human angiotensin–converting enzyme 2 in acute respiratory distress syndrome. Crit Care.

[32] Gu H, Xie Z, Li T, Zhang S, Lai C, Zhu P, Wang K, Han L, Duan Y, Zhao Z, Yang X, Xing L, Zhang P, Wang Z, Li R, Yu JJ, Wang X, Yang P (2020). Angiotensin-converting enzyme 2 inhibits lung injury induced by respiratory syncytial virus. Sci Rep.

[33] Gage JR, Fanarow G, Hamilton M, Widawski M, Martinez-Maza O, Vredevoe D (2004). Beta blockers and angiotensin-converting enzyme inhibitor therapy is associated with decreased TH1/Th2 cytokine ratios and inflammatory cytokines production in patients with chronic heart failure. Neuroimmunomodulator.

[34] Moher D, Liberati A, Tetzlaff J, Altman DG, The PRISMA Group (2009). Preferred Reporting Items for Systematic Reviews and Meta-Analysis: the PRISMA statement. PLoS Med.

[35] Ottawa Hospital Research Institute. https://www.ohri.ca/programs/clinical_epidemiology/oxford.asp. Assessed 9 June 2020.

[36] Mehta N, Kalra A, Nowacki AS, Anjewierden S, Han Z, Bhat P, Carmona-Rubio AE, Jacob M, Procop GW, Harrington S, Jehi L, Young JB, Chung MK (2020) Association of use of angiotensin-converting enzyme inhibitors and angiotensin II receptor blockers with testing positive for coronavirus disease 2019 (COVID-19). Jama Cardiol:e201855. 10.1001/jamacardio.2020.1855 10.1001/jamacardio.2020.1855 PMC7201375 32936273

[37] Reynolds HR, Adhikari S, Pulgarin C, Troxel AB, Iturrate E, Johnson SB, Hausvater A, Newman JD, Berger JS, Bangalore S, Katz SD, Fishman GI, Kunichoff D, Chen Y, Ogedegbe G, Hochman J (2020). Renin-angiotensin-aldolsterone inhibitors and risk of COVID-19. N Eng J Med..

[38] Rentsch CT, Kidwai-Khan F, Tate JP, Park LS, King JT, Skanderson M, Hauser RG, Schultze A, Javis CI, Holodniy M, Lo Re V 3rd, Akgun KM, Crothers K, Taddei TH, Freiberg MS, Justice AC (2020) COVID-19 testing, hospital admission, and intensive care among 2,026,227 United States veterans aged 54-75 years. Med Rxiv. 10.1101/2020.04.09.20059964 (Pre-print)

[39] Mancia G, Rea F, Ludergnani M, Apolone G, Carrao G (2020). Renini-angiotensin-aldolsterone system blockers and the risk of COVID-19. N Engl J Med..

[40] Elimination or prolongation of ACEI and ARB in Coronavirus disease 2019. https://clinicaltrials.gov. Accessed 30 May 2020.

[41] European Societies of Cardiology (2020). Position statement of ESC Council on Hypertension on ACE inhibitors and angiotensin receptor blockers.

[42] American Heart Association. HFSA/ACC/AHA Statement addresses concerns re: using RAAS antagonists in CoVID-19. https://professional.heart.org/professional/ScienceNews/UCM_505836_HFSAACCAHA-statement –addresses-concerns-re-using-RAAS-antagonists-in-COVID-19.jsp. Accessed 31 May 2020.

[43] Abajo FJ, Rodriguez-Martin S, Mejia-Abril G, Aguilar M, Garcia-Luque A, Laredo L, Laosa O, Centeno-Soto GA, Galvez MA, Puerro M, Gonzalez-Rojano E, Pedraza L, de Pablo I, Abad-Santos F, Rodriguez-Manas L, Gil M, Tobias A, Rodriguez-Miguel A, Rodriguez-Puyol D, MED-ACE2-COVID19 study group (2020). Use of renin-angiotensin-aldosterone system inhibitors and risk of COVID-19 requiring admission to hospital: a case-population study. Lancet.

[44] Bean DM, Kraljevic Z, Searle T, Bendayan R, O’Gallagher K, Pickles A, Folarin A, Roguski L, Noor K, Shek A, Zakeri R, Shah AJ, Teo JTH, Dobson RJB (2020) Treatment with ACE-inhibitors is associated with less severe disease with SARS-COVID-19 infection in a multi-site UK acute Hospital Trust. MedRxiv. 10.1101/2020.04.07.20056788 (Preprint) 10.1002/ejhf.1924 PMC7301045 32485082

[45] De Spiegeleer A, Bronselaer A, Teo JT, Byttebier G, De Tre G, Belmans L, Dobson R, Wynendaele E, Van De Wiele C, Vandaele F, Van Dijck D, Bean D, Fedson D, De Spirgeleer B (2020). The effects of ARBs, ACEIs and statins on clinical outcomes of COVID-19 in nursing home residents. J Am Med Directors Assoc.

[46] Li J, Wang Z, Chen J, Zhang H, Deng A (2020). Association of renin-angiotensin system inhibitors with severity or risk of death in patients with hypertension hospitalized for coronavirus disease 2019 infection in Wuhan, China. JAMA Cardiol.

[47] Liu Y, Huang F, Xu J, Yang P, Qin Y, Cao M, Wang Z, Li X, Zhang S, Ye L, Lv J, Wei J, Xie T, Gao H, Xu K, Wang F, Liu L, Jiang C (2020) Antihypertensive angiotensin II receptor blockers associated to mitigation of disease severity in elderly COVID-19 patients. Med Rxiv. 10.1101/2020.03.20.20039586v1 (Preprint)

[48] Meng J, Xiao G, Zhang J, He X, Ou M, Bi J, Yang R, Di W, Wang Z, Li Z, Gao H, Liu L, Zhang G (2020). Renin-angiotensin system inhibitors improve the clinical outcomes of COVID-19 patients with hypertension. Emerg Microbes Infect.

[49] Rossi PG, Marino M, Formisano D, Venturelli F, Grilli R (2020) Characteristics and outcomes of a cohort of SARS-CoV-2 patients in the province of Reggio Emilia, Italy. Med Rxiv. 10.1101/2020.04.13.20063545 10.1371/journal.pone.0238281 PMC7451640 32853230

[50] Yang G, Tan Z, Zhou L, Yang M, Peng L, Liu J, Cai J, Yang R, Han J, Huamg Y, He S (2020). Effects of angiotensin II receptor blockers and ACE (angiotensin converting enzyme) inhibitors on viral infection, inflammatory status and clinical outcomes in patients with COVID-19 and hypertension: a single-center study. Hypertension.

[51] Feng Y, Ling Y, Bai T, Xie Y, Huang J, Li J, Xiong W, Yang D, Chen R, Lu F, Lu Y, Liu X, Chen Y, Li X, Li Y, Summah HW, Lin H, Yan Y, Zhou M, Lu H, Qu J (2020). COVID-19 with different severities: a multicentre study of clinical features. Am J Respir Crit Care Med.

[52] Zhang P, Zhu L, Cai J, Lei F, Qin J, Xie J, Liu Y, Zhao Y, Huang X, Lin L, Xia M, Chen M, Cheng X, Zhang X, Guo D, Peng Y, Ji Y, Chen J, She Z, Wang Y, Xu Q, Tan R, Wang H, Lin J, Luo P, Fu S, Cai H, Ping Y, Xiao B, Mao W, Liu L, Yan Y, Liu M, Chen M, Zhang X, Wang X, Touz RM, Xia J, Zhang B, Huang X, Yuan Y, Loomba R, Liu PP, Li H (2020). Association of inpatient use of angiotensin converting enzyme inhibitors and angiotensin II receptor blockers with mortality among patients with hypertension hospitalized with COVID-19. Circ Res.

[53] Peng YD, Meng K, Guan HQ, Leng L, Zhu RR, Wang BY, He MA, Cheng LX, Huang K, Zeng QT (2020). Clinical characteristics and outcomes of 112 cardiovascular disease patients infected by 2019-nCoV. Zhonghua xin xue Guan Bing za zhi..

[54] Huang Z, Cao J, Yao Y, Jin X, Luo Z, Xue Y, Zhu C, Song Y, Wang Y, Zou Y, Qian J, Yu K, Gong H, Ge J (2020). The effects of RAS blockers on the clinical characteristics of COVID-19 patients with hypertension. Ann Transl Med.

[55] Zhang X, Yu J, Pan L, Jiang H (2020). ACEI/ARB use and risk of infection or severity or mortality of COVID-19: a systematic review and meta-analysis. Pharmacol Res.

[56] Guo X, Zhuo Y, Hong Y (2020). Decreased mortality of COVID-19 with renin-angiotensin-aldosterone system inhibitors therapy in patients with hypertension: a meta-analysis. Hypertension.

