# Supplementary material for: Risk of SARS-CoV-2 infection and COVID-19 prognosis with the use of renin–angiotensin–aldosterone system (RAAS) inhibitors: a systematic review
Source: Futur J Pharm Sci. 2021 Mar 24;7(1):73. doi: 10.1186/s43094-021-00224-4 (PMC7988641; doi:10.1186/s43094-021-00224-4)
Supplement: Supplementary file 1 — Additional file 1. Assessment of risks of bias for observational studies according to the Newcastle–Ottawa scale. [file 43094_2021_224_MOESM1_ESM.doc]

**Risk of SARS-CoV-2 infection, and COVID-19 prognosis with the use of Renin-Angiotensin-Aldosterone-System (RAAS) Inhibitors: A systematic review**

Chinonyerem O. Iheanacho^1^, email: nonye2m@yahoo.com; Valentine U. Odili^2^ email:vuodili@yahoo.com, Uchenna I.H Eze^3^, email: ifyeze3000@gmail.com

^1^Department of Clinical Pharmacy and Public Health, Faculty of Pharmacy, University of Calabar, Calabar, Nigeria

^2^Department of Clinical Pharmacy and Pharmacy Practice, University of Benin, Benin City, Nigeria

^3^Department of Clinical Pharmacy and Biopharmacy, Olabisi Onabanjo University, Sagamu, Nigeria

**Corresponding author**: Chinonyerem O. Iheanacho

**Postal address**: Department of Clinical Pharmacy and Public Health, Faculty of Pharmacy, University of Calabar, Calabar, Nigeria.

**Phone number**: +2348037923560

**Assessment of risks of bias for observational studies according to the Newcastle-Ottawa scale^1^**

**Table s1: Cohort studies**

| **Study** | **Selection bias** | **Comparability bias** | **Outcome bias** |
| --- | --- | --- | --- |
| Mehta *et al*, 2020 (USA)^2^ | 4/4 | 2/2 | 3/3 |
| Renyolds *et al,* 2020 (USA)^3^ | 4/4 | 1/2 | 3/3 |
| **#**Rentsch *et al,* 2020 (USA)^4^ | 4/4 | 2/2 | 3/3 |
| **#**Bean *et al*, 2020 (United Kingdom)^5^ | 4/4 | 1/2 | 3/3 |
| De Spiegeleer *et al,* 2020 (Belgium)^6^ | 4/4 | 2/2 | 3/3 |
| Li et al, 2020 (China)^7^ | 4/4 | 0/2 | 3/3 |
| **#**Liu *et al*, 2020 (China)^8^ | 4/4 | 0/2 | 3/3 |
| Rossi *et al,* 2020 (Italy)^9^ | 4/4 | 2/2 | 3/3 |
| Feng *et al*, 2020 (China)^10^ | 4/4 | 0/2 | 3/3 |
| Zhang et al, 2020^11^ | 4/4 | 2/2 | 3/3 |
| Guo et al, 2020^12^ | 4/4 | 2/2 | 3/3 |

**#**non-peer reviewed (as at the time of preparing this manuscript)

The numerators denote the number of stars and the denominators denote the maximum number of stars for each category. A higher number of stars indicate a lower risk of bias.

**Table s2: Case-control studies**

| **Study** | **Selection bias** | **Comparability bias** | **Exposure** |
| --- | --- | --- | --- |
| Mancia et al, 2020 (Italy)^13^ | 4/4 | 2/2 | 3/3 |
| Abajo *et al*, 2020 (Spain)^14^ | 4/4 | 2/2 | 3/3 |
| Meng *et al*, 2020 (China)^15^ | 4/4 | 0/2 | 3/3 |
| Yang *et al*, 2020 (China)^16^ | 4/4 | 2/2 | 3/3 |
| Zhang *et al*, 2020 (China)^17^ | 4/4 | 2/2 | 3/3 |
| Peng et al, 2020 (China)^18^ | 4/4 | 0/2 | 3/3 |
| Huang et al, 2020 (China)^19^ | 3/4 | 1/2 | 3/3 |

The numerators denote the number of stars and the denominators denote the maximum number of stars for each category. A higher number of stars indicate a lower risk of bias.

**References**

1. Ottawa Hospital Research Institute. https://www.ohri.ca/programs/clinical_epidemiology/oxford.asp. Assessed June 9, 2020.
2. Mehta N, Kalra A, Nowacki AS, Anjewierden S, Han Z, Bhat P, Carmona-Rubio AE, Jacob M, Procop GW, Harrington S, Jehi L, Young JB, Chung MK (2020) Association of use of angiotensin-converting enzyme inhibitors and angiotensin11 receptor blockers with testing positive for coronavirus disease 2019 (COVID-19). Jama Cardiol e201855. [Doi: 10.1001/jamacardio.2020.1855](https://doi.org/10.1001/jamacardio.2020.1855).
3. Reynolds HR, Adhikari S, Pulgarin C, Troxel AB, Iturrate E, Johnson SB, Hausvater A, Newman JD, Berger JS, Bangalore S, Katz SD, Fishman GI, Kunichoff D, Chen Y, Ogedegbe G, Hochman J (2020) Renin-angiotensin-aldolsterone inhibitors and risk of COVID-19. N Eng J Med. Doi: 10.1056/NEJMoa2008975.
4. Rentsch CT, Kidwai-Khan F, Tate JP, Park LS, King JT, Skanderson M, Hauser RG, Schultze A, Javis CI, Holodniy M, Lo Re lll V, Akgun KM, Crothers K, Taddei TH, Freiberg MS, Justice AC (2020) COVID-19 testing, hospital admission, and intensive care among 2,026,227 United States veterans aged 54-75 years. Med Rxiv. Doi:10.1101/2020.04.09.20059964. (Pre-print)
5. Mancia G, Rea F, Ludergnani M, Apolone G, Carrao G (2020) Renini-angiotensin-aldolsterone system blockers and the risk of COVID-19. N Engl J Med. Doi: 10.1056/NEJMoa2006923.
6. Abajo FJ, Rodriguez-Martin S, Mejia-Abril G, Aguilar M, Garcia-Luque A, Laredo L, Laosa O, Centeno-Soto GA, Galvez MA, Puerro M, Gonzalez-Rojano E, Pedraza L, de Pablo I, Abad-Santos F, Rodriguez-Manas L, Gil M, Tobias A, Rodriguez-Miguel A, Rodriguez-Puyol D, MED-ACE2-COVID19 study group (2020) Use of rennin-angiotensin-aldosterone system inhibitors and risk of COVID-19 requiring admission to hospital: a case-population study. Lancet 395(10238): 1705-1714. Doi: 10.1016/S0140.
7. Bean DM, Kraljevic Z, Searle T, Bendayan R, O’Gallagher K, Pickles A, Folarin A, Roguski L, Noor K, Shek A, Zakeri R, Shah AJ, Teo JTH, Dobson RJB (2020) Treatment with ACE-inhibitors is associated with less severe disease with SARS-COVID-19 infection in a multi-site UK acute Hospital Trust. MedRxiv. [Doi](https://doi/): 10.1101/2020.04.07.20056788. (Preprint)
8. De Spiegeleer A, Bronselaer A, Teo JT, Byttebier G, De Tre G, Belmans L, Dobson R, Wynendaele E, Van De Wiele C, Vandaele F, Van Dijck D, Bean D, Fedson D, De Spirgeleer B (2020) The effects of ARBs, ACEIs and statins on clinical outcomes of COVID-19 in nursing home residents. J American Med Directors Assoc 21:909-914. Doi:10.1101/j.jamda.2020.06.018.
9. Li J, Wang Z, Chen J, Zhang H, Deng A (2020) Association of Renin-angiotensin system inhibitors with severity or risk of death in patients with hypertension hospitalized for coronavirus disease 2019 infection in Wuhan, China. JAMA Cardiology. Doi:10.1001/jamacardio.2020/624.
10. Liu Y, Huang F, Xu J, Yang P, Qin Y, Cao M, Wang Z, Li X, Zhang S, Ye L, Lv J, Wei J, Xie T, Gao H, Xu K, Wang F, Liu L, Jiang C (2020) Antihypertensive angiotensin 11 receptor blockers associated to mitigation of disease severity in elderly COVID-19 patients. Med Rxiv. Doi:10.1101/2020.03.20.20039586v1. (Preprint)
11. Meng J, Xiao G, Zhang,,He X, Ou M, Bi J, Yang R, Di W, Wang Z, Li Z, Gao H, Liu L, Zhang G (2020) Renin-angiotensin system inhibitors improve the clinical outcomes of COVID-19 patients with hypertension. Emerg Microbes Infect 9:757-760.
12. Rossi PG, Marino M, Formisano D, Venturelli F, Grilli R (2020) Characteristics and outcomes of a cohort of SARS-CoV-2 patients in the province of Reggio Emilia, Italy. Med Rxiv. Doi:10.1101/2020.04.13.20063545.
13. Yang G, Tan Z, Zhou L, Yang M, Peng L, Liu J, Cai J, Yang R, Han J, Huamg Y, He S (2020) Effects of angiotensin 11 receptor blockers and ACE (Angiotensin Converting Enzyme) inhibitors on viral infection, inflammatory status and clinical outcomes in patients with COVID-19 and hypertension: a single-center study. Hypertension 76(1): 51 -58. Doi: 10.1161/HYPERTENSIONAHA.120.15143.
14. Feng Y, Ling Y, Bai T, Xie Y, Huang J, Li J, Xiong W, Yang D, Chen R, Lu F, Lu Y, Liu X, Chen Y, Li X, Li Y, Summah HW, Lin H, Yan Y, Zhou M, Lu H, Qu J (2020) COVID-19 with different severities: A multicentre study of clinical features. Am J Respir Crit Care Med 201(11): 1380-1388. Doi:10.1164/rccm.202002-0445OC.
15. Zhang P, Zhu L, Cai J, Lei F, Qin J, Xie J, Liu Y, Zhao Y, Huang X, Lin L, Xia M, Chen M, Cheng X, Zhang X, Guo D, Peng Y, Ji Y, Chen J, She Z, Wang Y, Xu Q, Tan R, Wang H, Lin J, Luo P, Fu S, Cai H, Ye Ping, Xiao B, Mao W, Liu L, Yan Y, Liu M, Chen M, Zhang X, Wang X, Touz RM, Xia J, Zhang B, Huang X, Yuan Y, Loomba R, Liu PP, Li H (2020) Association of inpatient use of angiotensin converting enzyme inhibitors and angiotensin 11 receptor blockers with mortality among patients with hypertension hospitalized with COVID-19. Circ Res 127(6): e147. Doi:10.1161/CIRCRESAHA.120.317134.
16. Peng YD Meng K, Guan HQ, Leng L, Zhu RR, Wang BY, He MA, Cheng LX, Huang K, Zeng QT (2020) Clinical characteristics and outcomes of 112 cardiovascular disease patients infected by 2019-nCoV. Zhonghua xin xue Guan Bing za zhi. 48(0):E004. Doi:10.3760/cma.j.cn112148-20200220-00105.
17. Huang Z, Cao J, Yao Y, Jin X, Luo Z, Xue Y, Zhu C, Song Y, Wang Y, Zou Y, Qian J, Yu K, Gong H, Ge J (2020) The effects of RAS blockers on the clinical characteristics of COVID-19 patients with hypertension. Ann Transl Med 8(7): 430. [Doi:10.21037/atm.2020.03.229](https://doi.org/10.21037/atm.2020.03.229).
18. Zhang X, Yu J, Pan L, Jiang H (2020) ACEI/ARB use and risk of infection or severity or mortality of COVID-19: A systematic review ad Meta-analysis. Pharmacol Res 104927. Doi:10.1016/j.phrs.2020.104927.
19. Guo X, Zhuo Y, Hong Y (2020) Decreased mortality of COVID-19 with rennin-angiotensin-aldosterone system inhibitors therapy in patients with hypertension: A meta-analysis. Hypertension 76:e13-e14. Doi:HYPERTENSIONAHA.120.15572.
